# ADSCs Combined with Melatonin Promote Peripheral Nerve Regeneration through Autophagy

**DOI:** 10.1155/2022/5861553

**Published:** 2022-07-20

**Authors:** Ziqiang Zhang, Mengyu Zhang, Zhixiang Zhang, Yingying Sun, Jiajia Wang, Chenhao Chang, Xinyan Zhu, Monan Li, Yumei Liu

**Affiliations:** ^1^College of Animal Science and Technology, Henan University of Science and Technology, Luoyang, Henan 471000, China; ^2^College of Life Science, Yangtze University, Jingzhou, Hubei 434023, China; ^3^School of Materials Science and Engineering, Henan University of Science and Technology, Luoyang, Henan 471000, China

## Abstract

**Background:**

In the early stage of nerve injury, damaged tissue is cleared by autophagy. ADSCs can promote nerve axon regeneration. However, the microenvironment of the injury was changed, and ADSCs are easily apoptotic after transplantation. Mel plays a role in the apoptosis, proliferation, and differentiation of ADSCs. Therefore, we investigated whether Mel combined with ADSCs promoted peripheral nerve regeneration by enhancing early autophagy of injured nerves.

**Materials and Methods:**

SD rats were randomly split into the control group, model group, Mel group, ADSCs group, ADSCs + Mel group, and 3-MA group. On day 7, autophagy was observed and gait was detected on days 7, 14, 21, and 28. On the 28th day, the sciatic nerve of rats' renewal was detected.

**Results:**

After 1 w, compare with the model group, the number of autophagosomes and lysosomes and the expressions of protein of LC3-II/LC3-I and Beclin-1 in the ADSCs + Mel group were prominently increased, while the 3-MA group was significantly decreased. After 4 w, the function of the sciatic nerve in ADSCs + Mel was similar to that in the control group. Compared with the model group, the ADSCs + Mel group significantly increased myelin regeneration and the number of motor neurons and reduced gastrocnemius atrophy.

**Conclusions:**

It was confirmed that ADSCs combined with Mel could promote sciatic nerve regeneration in rats by changing the early autophagy activity of the injured sciatic nerve.

## 1. Introduction

The peripheral nervous system (PNS) and central nervous system (CNS) are the two parts of the nervous system [1]. The CNS is secured by the blood-brain barrier. In contrast, the PNS is disclosed to mechanical trauma, inflammatory processes, and toxins, causing easy injury [2]. Among these injuries, mechanical damage is currently the most important factor [3]. Notably, peripheral nerve injury may lead to motor and sensory dysfunction and irreversible tissue atrophy [4]. As we all know, axons have competence to regenerate, but nerve recovery after PNI is frequently slow and there is no first-rank treatment [5, 6].

In the early period of peripheral nerve injury (PNI), impaired organelles, cellular debris, and dysfunctional proteins pile up at the injured site in a short period, bringing about partial stress that obstructs the capability of Schwann cells (SCs) to motivate nerve regeneration [7, 8]. Therefore, the fast-speed clearance of impaired organelles, cellular debris, and dysfunctional proteins are playing a vital part in axon repair and functional recovery.

Autophagy is a conserved intracellular mechanism for maintaining cellular homeostasis in which impaired organelles, cellular debris, and dysfunctional proteins are degraded [9, 10]. In addition, a study showed that a moderate amount of autophagy in SCs after PNI could motor function reconstruction and promote nerve regeneration [7]. At the beginning of Wallerian degeneration, the autophagy of SCs plays an important role in a series of pathophysiological changes after PNI [3]. Thus, autophagy plays an important role in the development and functional maintenance of PNI.

Previous studies have confirmed the conducive influence of mesenchymal stem cells (MSCs) for facilitating nerve regeneration [11, 12]. ADSCs, a source of MSCs, can be induced to no-mesenchymal fates in in vitro, such as neurons, astrocytes, and SCs, to support nerve regeneration [13, 14]. Multiple types of research have confirmed the capacity of ADSCs to promote nerve regeneration in animal models of an injured peripheral nerve [12, 15–18]. However, it is not clear whether ADSCs promote myelination by promoting SCs autophagy after nerve injury [10]. Although the use of cells could improve PNI, engrafted stem cells confront low cell survival rates because of oxidative stress and the hostile microenvironment of the injured body, a further effective strategy in cell-based therapies appear to be necessary [19]. Research has shown that pharmacological preconditioning of stem cells is a reasonable way to strengthen their defenses against damage in the injury environment [20]. Melatonin (Mel), a pineal gland hormone, has been demonstrated to play a major part in adjusting the pathological process [21]. Previous studies have used an intraperitoneal injection of Mel to treat PNI and showed good therapeutic effects [22–24]. Another study has been reported of Mel directly injected into the lesion to improve the treatment. Stavisky et al. have demonstrated that injecting 100 *μ*M Mel into the lesion, it can increase the ability to PEG-fuse sciatic axons in vivo [25]. What is more, Mel has been shown to exert a vital function in the survival, differentiation, migration, and proliferation of cells [21]. Moreover, MSCs pretreated with Mel more withstand to oxidative stress injury [19, 26].

Therefore, this study aims to investigate the effects of ADSCs combined with Mel on autophagy in SCs and can effectively promote nerve regeneration after being injected into the injury site.

## 2. Materials and Methods

### 2.1. Isolation and Culture of ADSCs

Briefly, the inguinal fat pad was removed from the abdominal cavity of rats and anesthetized with diethyl ether. The adipose tissue was digested at 37°C for 70 min using type I collagenase (Sigma, USA). After centrifugation at 170 × *g* for 10 min, the cells were resuspended in the lower layer using DMEM/F12 (HyClone, USA) and then filtrated through a 70 *μ*m mesh. The collected cells were cultured in a 37°C, 5% CO_2_ incubator in DMEM/F12 containing 10% FBS (Gibco, USA). After 48 h, the medium was changed for the first time. When the cells reached confluence, the adherent cells were detached with trypsin/ethylenediaminetetraacetic acid (EDTA) (Sigma, USA) and reseeded for expansion [27, 28].

### 2.2. Multidifferentiation Ability of ADSCs

The ADSCs were seeded into a 6-well plate at a density of 5 × 10^4^ cells/ml. When the cells reached approximately 80% confluence, the medium was replaced by either adipogenic inducer medium or osteogenic inducer medium. The adipogenic inducer medium consisted of DME/F12 containing 1×10^−6^ mol/L dexamethasone (Sigma, USA), 10 mg/L insulin (Sigma, USA), 0.5 mmol/L 3-isobutyl-1-methylxanthine (Solarbio, Shanghai, China), and 0.2 mmol/L indomethacin (Solarbio, Shanghai, China). The osteogenic inducer medium is DMEM/F12 containing 1 × 10^−6^ mol/L dexamethasone (Sigma, USA), 50 *μ*g/mL ascorbic acid (Solarbio, Shanghai, China), and 10 mmol/L *β*-sodium glycerophosphate (Solarbio, Shanghai, China). The inducer medium was changed every 3 d. After 15 d of induction, the cells were tested for the conversion of adipocytes with oil red O staining solution (Sigma, USA). After 21 d of induction, cells were stained with alizarin red staining (Ybscience, Shanghai, China) solution to detect the conversion to bone cells [29].

### 2.3. Materials

Adult male SD rats weighing 180–220 g (*n* = 12) were obtained from the Medical Experimental Animal Center, Henan University of Science and Technology. All experimental processes were agreed by the Institutional Animal Care and Use Committee of Henan University of Science and Technology (China) (No. 20190319087).

### 2.4. Drug Configuration

**Figure 1:**
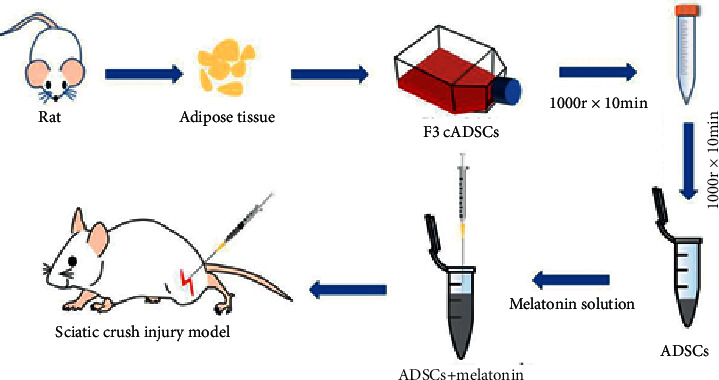
Drug allocation process.

Mel, bought from Sigma Aldrich (St. Louis, MO, USA), was deliquesced in thyl alcohol (Mel solvent) and diluted with PBS. The final thyl alcohol concentration reached to be 5%, while the concentration of Mel is 50 *μ*M. This solution needs to be prepared on-site service and stored in the dark. In the next step, the centrifuged ADSCs are suspended in 50 *μ*M Mel and the ADSC concentration of 1 × 105/*μ*l (Figure 1).

### 2.5. Induction of Peripheral Nerve Injury

**Figure 2:**
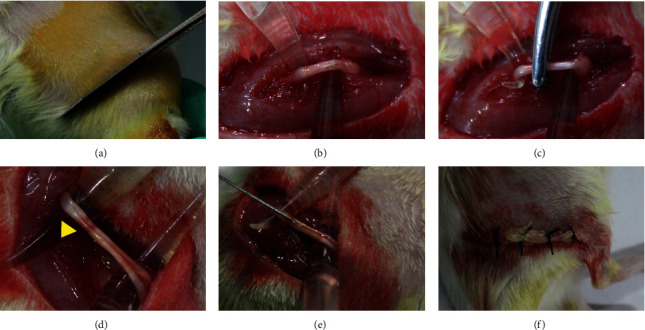
ADSCs therapy process. (a)–(d) The sciatic nerve was crushed with tweezers. (e) The ADSCs were injected. (f) Postoperative suture. The lesion appears transparent (yellow arrows).

The SD rats were anesthetized with pentobarbital (50 mg/kg), and exposed the right sciatic nerve. Next, the nerve was tightly crushed by hemostatic forceps three times for 10 s each at 10 s intervals [11]. Then, we can see its color became translucent. This procedure causes all axons of the sciatic nerve where entire transection is, but the epineurium is intact (Figure 2).

### 2.6. Treatment

**Figure 3:**
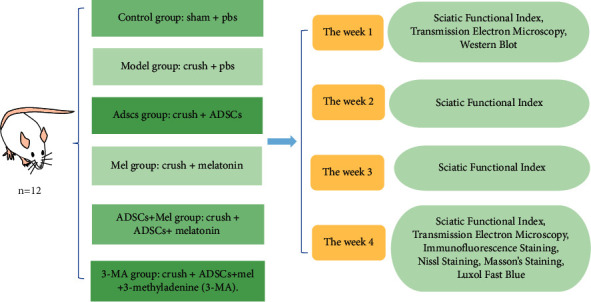
Grouping of experiments.

Animals were stochastic divided into six groups (*n* = 12) (Figure 3): control group: sham + PBS, model group: crush + PBS, ADSCs group: crush + ADSCs, Mel group: crush + Mel, ADSCs + Mel group: crush + ADSCs + Mel, and 3-MA group: crush + ADSCs + Mel+3-methyladenine (3-MA). The dose of the solution in each group was 10 *μ*l. Next, ADSCs and Mel were transplanted with microliter syringes in the intralesional. After this step, the wound was closed by suture. 3-Ma produced by Sigma Aldrich (St. Louis, MO, USA) is dissolved in 0.9% NaCl. Experimental animals (3-MA group) received a dose of 50 mg/kg 3-MA daily with intraperitoneal injections for 7 d after the surgery.

### 2.7. Sciatic Functional Index (SFI)

The week one, two, three, and four postsurgery, the walking pattern of animals was recorded for the analysis of the sciatic functional index (SFI). Concisely, a cardboard box with open ends (long = 50 cm, wide = 10 cm, and high = 15 cm) was made. Put a piece of white paper (10 × 50 cm) in the cardboard box and gently rub the ink on the bottom of the rat's feet with a brush. The rat was then placed at the entrance of the cardboard box, and rats would walk straight through the cardboard box and leave footprints on the white paper. The lengths of the first to the fifth toe (TS), the second toe to the fourth toe (IT), and the third toe to its heel (PL) were measured on the contralateral normal side (*N*) and the experimental side (*E*). The SFI value was calculated automatically according to the following modified formula:

(1) $$\begin{array}{c} \text{SFI }=\frac{-38.3\left(\text{EPL}–\text{NPL}\right)}{\text{NPL}}+\frac{109.5\left(\text{ETS }–\text{ NTS}\right)}{\text{NTS}}+\frac{13.3\left(\text{EIT}-\text{NIT}\right)}{\text{NITT}}-8.8. \end{array}$$

An SFI value of −100 meant total impairment, whereas 0 was considered normal meant total impairment.

### 2.8. Western Blot Analysis

After 7 d of surgery, a nerve segment of injury was removed to collect total proteins. Sciatic nerve tissues were uniformized in lysis buffer (80 mM *β*-glycerophosphate; 50 mM Tris-HCl, pH7.5; 150 mM NaCl; 1% Triton X-100; 2 mM EDTA; 25 mM NaPPi; and 0.2 mM Na_3_VO_4_) that contained a protease inhibitor cocktail and centrifuged. The protein concentration in the supernatant was determined with a BCA protein assay kit (Servicebio, Wuhan, China). Proteins (20 *μ*g) were resolved by 15% acrylamide gel and transferred onto a PVDF membrane on the ice. The membrane was blocked with 5% skim milk in 0.5% TBST and incubated with primary antibody rabbit anti-Beclin-1 (1 : 1,000; Servicebio, Wuhan, China) and rabbit anti-LC3 (1 : 1,000; Servicebio, Wuhan, China) at 4°C. Next, wash with TBST and incubate with horseradish peroxidase (HRP) conjugated secondary antibodies (1 : 10000) at room temperature and develop using enhanced chemiluminescent (ECL). *β*-Actin (mouse anti-*β*-actin, 1 : 1,000; Servicebio, Wuhan, China) was used as an internal reference. The intensity of protein bands was visualized using a computer image analysis system (Alpha Ease FC).

### 2.9. Immunofluorescent Assay of Regenerated Nerves

At 4 w postoperatively, the distal end of injured nerves was harvested after SD rats were sacrificed and fixed by 4% paraformaldehyde for 24 h, embedded in paraffin, and mounted on polylysine precoated slides. The nerve tissue paraffin sections were blocked and labeled with rabbit anti-rat S-100 (red) (1 : 200; Sanying, Wuhan, China), rabbit anti-rat myelin basic protein (MBP) (red) (1 : 500; Sanying, Wuhan, China), and rabbit anti-rat NF-200 (green) (1 : 100; Sanying, Wuhan, China). The sections were incubated with sheep anti-rabbit IgG Cy3-conjugated secondary antibody and then mounted with a mounting medium containing 4,6-diamidino-2-phenylindole (DAPI). The S-100/MBP/NF-200-positive cells were figured in three random fields for each section of rats in each group.

### 2.10. Histological Assessment of Regenerated Nerve

Following 1 and 4 w of surgery, sciatic nerve samples were prefixed with 2.5% glutaraldehyde at 4°C overnight. Following, the nerves were postfixed in 1% osmium tetroxide for 2 h at 4°C. Then, tissues were dehydrated with ethanol acetone at 4°C and were embedded in Epon812 ethoxylate resin. After these processes, the sections of 60 *μ*m thickness were observed under a transmission electron microscope (Japan Electron Optics Laboratory Co., Ltd., Japan).

### 2.11. Luxol Fast Bule

On the week four, rats were humanely euthanatized and nerves immersed in 4% paraformaldehyde for 48 h and paraffin-embedded. The sections of the nerve were deparaffinized in xylene and hydrated in 100% ethanol, 85% ethanol, and 85% ethanol for 5 min each and finally rinsed in PBS. Next, the sections were stained overnight at 56°C in 0.1% Luxol Fast Blue (LFB) (Sigma, St. Louis, MO, USA) in acidified 95% ethanol and then rinsed in 95% ethanol and differentiated in 95% ethanol.

### 2.12. Gastrocnemius Muscle

After the nerve samples were removed, the gastrocnemius muscles of the operated and normal hind legs of rats in each group were stripped and weighed. The gastrocnemius wet weight ratio was calculated according to the formula: surgical side/normal side*∗*100%. Then, the gastrocnemius muscle belly samples were fixed in 4% paraformaldehyde for 24 h, embedded in paraffin cut into 5 mm sections, and stained with Masson's collagen staining and H&E staining method. Each sample was imaged with five random regions for the collagen fiber percentage analysis using Image-Pro Plus.

### 2.13. Nissl Staining

The spinal cord of rats was collected 28 days after surgery. The spinal cord tissues collected from each rat were sliced into 5 *µ*m sections, place them for 30 min at room temperature, immersed in xylene, 100% ethanol, 75% ethanol, and distilled water for 3 min each, and then stain with a 1% toluidine blue solution at 56°C for 20 min. The sections were douched in distilled water and differentiated in 1% glacial acetic acid for 2 min, immersed in 95% ethanol, and soak in anhydrous ethanol, anhydrous ethanol, xylene, and neutral gum, and look at a microscope. The Nissl bodies for Nissl staining were then analyzed using Image-Pro Plus 6.0 software (Media Cybernetics, Rockville, MD).

### 2.14. Statistical Analysis

Data were analyzed with SPSS 20 through one-way ANOVA followed by Tukey's post hoc multiple comparison test. Results were presented as mean SD. *P* < 0.05 was considered a statistically significant threshold of differences.

## 3. Result

### 3.1. Characterization of Rat ADSCs

**Figure 4:**
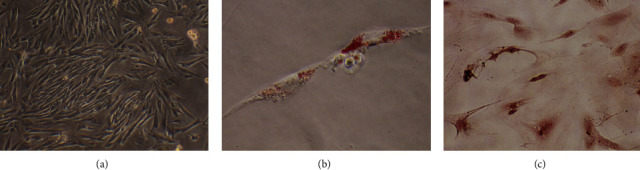
Characterization of adipose tissue-derived mesenchymal stem cells (ADSCs). (a) In passage three, the ADSCs exhibited a spindle-shaped fibroblastic morphology (magnification, ×100). (b) After dyeing with oil red O, the lipid droplets in the cells were stained red (magnification, ×400). (c) After staining with alizarin red dye, calcified nodules in cells were stained black (magnification, ×400).

At passage three, the cultured rat ADSCs exhibited a fibroblast-like, long shuttle morphology (Figure 4(a)). Positive alizarin red and oil red O staining confirmed that the cells could successfully differentiate into bone cells and adipocytes, respectively (Figures 4(b) and 4(c)). The ADSCs exhibited the potential for osteogenic and adipogenic differentiation following culture in osteogenic and adipogenic growth media.

### 3.2. ADSCs Combined with Mel Can Promote Sciatic Nerve Function Recovery

**Figure 5:**
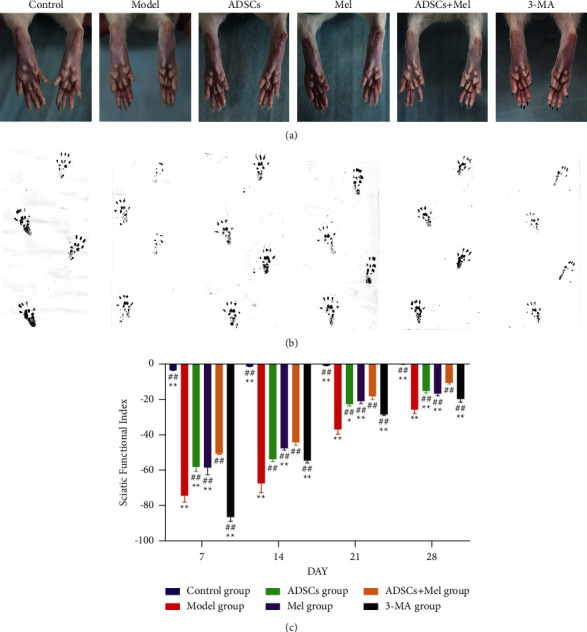
Effects of ADSCs combined with Mel on functional recovery. (a) Recovery of foot morphology. (b) Rat footprint. (c) SFI scores for the functional recovery at different time points. The control group, ADSCs group, Mel group, ADSCs + Mel group, and 3-MA group were compared with the model group, respectively. Each value is the mean ± SE of five independent determinations. ^##^*P* < 0.01 and ^#^*p* < 0.05, compared with the model group;  ^*∗*^ ^*∗*^*p* < 0.05, the control group, ADSCs group, model group, Mel group, ADSCs + Mel group, and 3-MA group were compared with the ADSCs + Mel group.

To verify the recovery of the rat's sciatic nerve after injury, SFI was computed by the Bain formula. The one result of feet and footprints in each group is shown in Figure 5. According to the SFI measurements shown in Figure 4, at the surgical operation of 1 w, in contrast to the control group, the SFI of the model group was particularly reduced by 21.106 times (*p* < 0.01), indicating limb dysfunction following nerve injury. We can see that on the one hand, the SFI values subsequently exhibited time-dependent increases; on the other hand, at 1, 2, 3, and 4 w after operation, the neurological function recovery of rats in treatment groups was higher than that of rats in the mode group (*p* < 0.01). At 4 w after treatment, the SFI values of the ADSCs + Mel group showed notably better recovery of regenerated nerve compared to Mel and ADSCs groups (*p* < 0.01), but inferior to the control group (*p* < 0.01). So, these results indicate that ADSCs and Mel treatment speeded up functional recovery of the sciatic nerve of rats after injury and the renewal of nerve function was faster in the ADSCs + Mel group than in Mel and ADSCs groups. However, when the autophagy inhibitor 3-MA was used, the effects of the combination therapy are extremely reduced (*p* < 0.01).

### 3.3. Effects of ADSCs Combined with Mel on Autophagy Activity after Sciatic Nerve Injury in Rats

**Figure 6:**
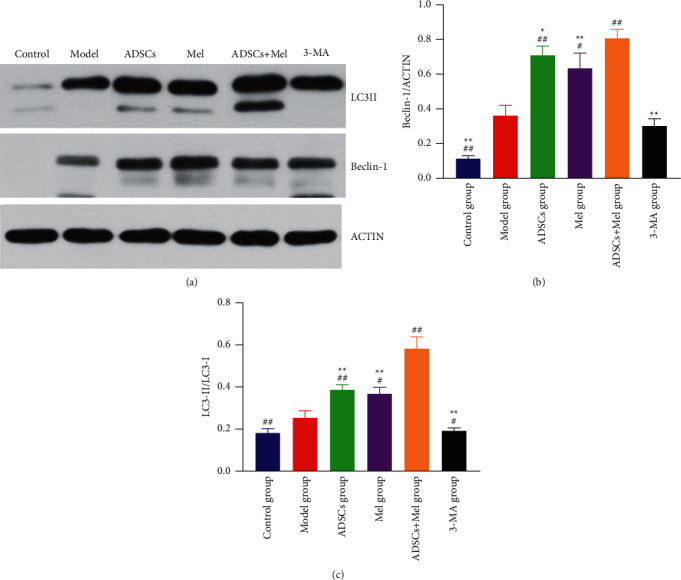
Expression of LC3 and Beclin-1 in rat sciatic nerve. (a) Western blot image of autophagy-related proteins LC3 and Beclin-1. (b) Beclin-1/ACTIN statistics. (c) LC3-II/LC3-I statistics. The control group, ADSCs group, Mel group, ADSCs + Mel group, and 3-MA group were compared with the model group, respectively. Each value is the mean ± SE of four independent determinations. ^##^*P* < 0.01, ^#^*p* < 0.05 compared with the model group;  ^*∗*^ ^*∗*^*p* < 0.05, the control group, ADSCs group, model group, Mel group, ADSCs + Mel group, and 3-MA group were compared with the ADSCs + Mel group.

To check into the important part of autophagy in nerve repair, the autophagy biomarkers, LC3 protein (LC3-I and LC3-II), and Beclin-1 were spotted by applicating the Western blot assay 7 days after surgery. Quantitative analysis shows that the model group striking increased the ratio of LC3-II to LC3-I and the protein level of Beclin-1 in contrast to the control group (*p* < 0.01, Figure 6). When compared to the model group, Beclin-1 in the ADSCs group, Mel group, and ADSCs + Mel group tended to increase (*p* < 0.01). In addition, the result revealed that Mel-treated ADSCs exhibited a higher ratio of LC3-II to LC3-I and the raised protein level of Beclin-1, compared with the only using cells or Mel (*p* < 0.01), while treatment of 3-MA reversed the trend (*p* < 0.01). Conversely, inhibiting autophagy using 3-MA adenine strongly inhibited the formation of autophagosomes.

**Figure 7:**
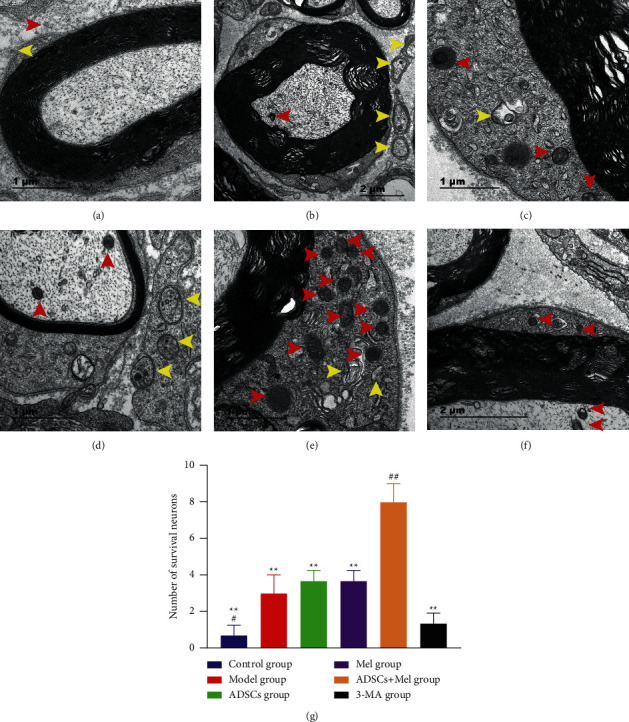
The mutations of autophagosomes on the 7th day after the sciatic nerve injury in rats were observed by electron microscopy. (a) Control group. (b) Model group. (c) ADSCs group. (d) Mel group. (e) Mel + ADSCs group. (f) 3-MA group. There are autophagosomes (yellow arrows) and lysosomes (red arrows) in the sciatic nerve. (g) Number of autophagosome statistics. The control group, ADSCs group, Mel group, ADSCs + Mel group, and 3-MA group were compared with the model group, respectively. Each value is the mean ± SE of four independent determinations. ^##^*P* < 0.01 and ^#^*p* < 0.05 compared with the model group;  ^*∗*^ ^*∗*^*p* < 0.05, the control group, ADSCs group, model group, Mel group, ADSCs + Mel group, and 3-MA group were compared with the ADSCs + Mel group.

The results of autophagosome observation by transmission electron microscopy (TEM) are shown in Figure 7. All groups had some autophagosomes compared to the control group, and no signs of autophagy were spotted in the control group and a greater lot of autophagosomes appear in ADSCs + Mel groups (Figure 7).

### 3.4. ADSCs Combined with Mel Enhances Axonal Regeneration and Increases Remyelination of Damaged Nerves

**Figure 8:**
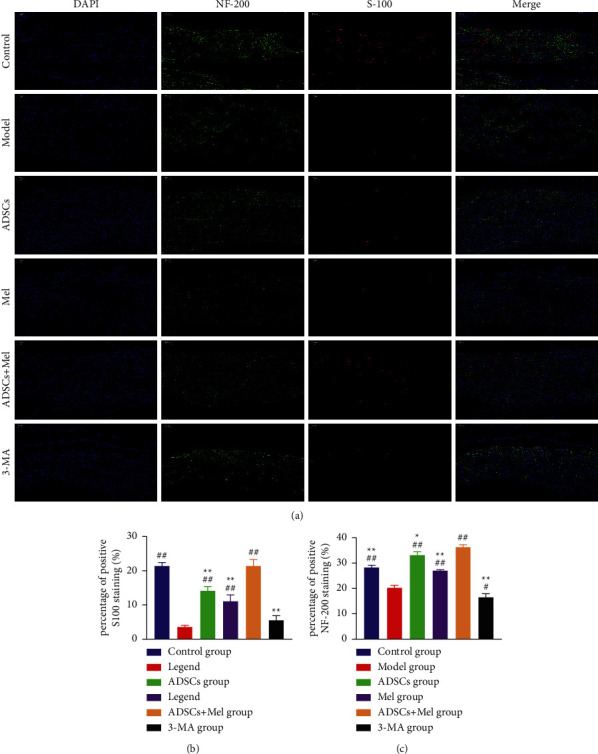
The expression of S-100 and NF-200 was detected by immunocytochemistry. (a) In experimental groups, the fluorescence intensity of NF-200 (green) and S-100 (red) elevated, which was detected by immunocytochemistry. (b)-(c) The fluorescence intensity. The control group, ADSCs group, Mel group, ADSCs + Mel group, and 3-MA group were compared with the model group, respectively. Each value is the mean ± SE of five independent determinations. ^##^*P* < 0.01 and ^#^*p* < 0.05, compared with the model group;  ^*∗*^ ^*∗*^*p* < 0.05, the control group, ADSCs group, model group, Mel group, ADSCs + Mel group, and 3-MA group were compared with the ADSCs + Mel group; scale bar = 100 *μ*m.

The repair effect of the regenerated nerve was evaluated by detecting the expression of neurofilament (NF)-200 and protein-100 (S-100) in the sciatic nerve. Immunofluorescence staining of rat sciatic nerve is shown in Figure 8. Therefore, we assessed whether ADSCs + Mel can increase the regeneration of axons and enhance the remyelination of damaged nerves. At 4 w postinjury, compared with the model group, S-100 and NF-200 immunoreactivity was prominently improved in all treatment groups. More importantly, at the 4 w after surgery, S-100 and NF-200 immunoreactivities were 4.868 and 0.393-fold higher, separately, in the control group as compared to the model group. The average percentage of fluorescence staining intensity of NF-200 and S-100 in the ADSCs + Mel group was higher than that in the ADSCs and Mel group (*p* < 0.01). The average percentage of fluorescence staining intensity of NF-200 and S-100 in the ADSCs + Mel group was higher than that in the 3-MA group.

**Figure 9:**
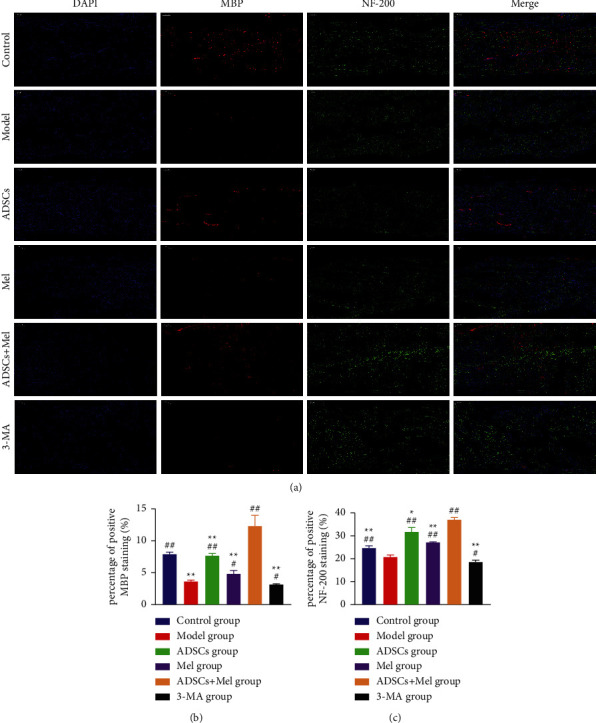
The immunofluorescence intensity expression of MBP and NF-200 was detected by immunocytochemistry. (a) In experimental groups, the fluorescence intensity of MBP (red) and NF-200 (green) elevated, which was detected by immunocytochemistry. (b)-(c) The fluorescence intensity was measured by integrated density pixel. The control group, ADSCs group, Mel group, ADSCs + Mel group, and 3-MA group were compared with the model group, respectively. Each value is the mean ± SE of five independent determinations. ^##^*P* < 0.01 and ^#^*p* < 0.05, compared with the model group;  ^*∗*^ ^*∗*^*p* < 0.05, the control group, ADSCs group, model group, Mel group, ADSCs + Mel group, and 3-MA group were compared with the ADSCs + Mel group; scale bar = 100 *μ*m.

MBP is a crucial component of myelin sheath in the PNS, and the expression of NF-200 is major for axon growth. Four weeks after surgery, NF-200 and MBP immunofluorescence intensity was 0.775 and 2.380-fold higher, respectively, in the control group of rats as contrasted to those subjected to injury but untreated. The expression of nerve regenerative proteins MBP and NF-200 at 4 w after injury was higher in the ADSCs + Mel group compared with the ADSCs or Mel group (*p* < 0.01; Figure 9). On the side, MBP and NF-200 immunofluorescence intensity expression was lower in the 3-MA group relative to the ADSCs + Mel group (*p* < 0.01).

### 3.5. Effects of ADSCs Combined with Mel on Myelin Regeneration after Sciatic Nerve Injury in Rats

**Figure 10:**
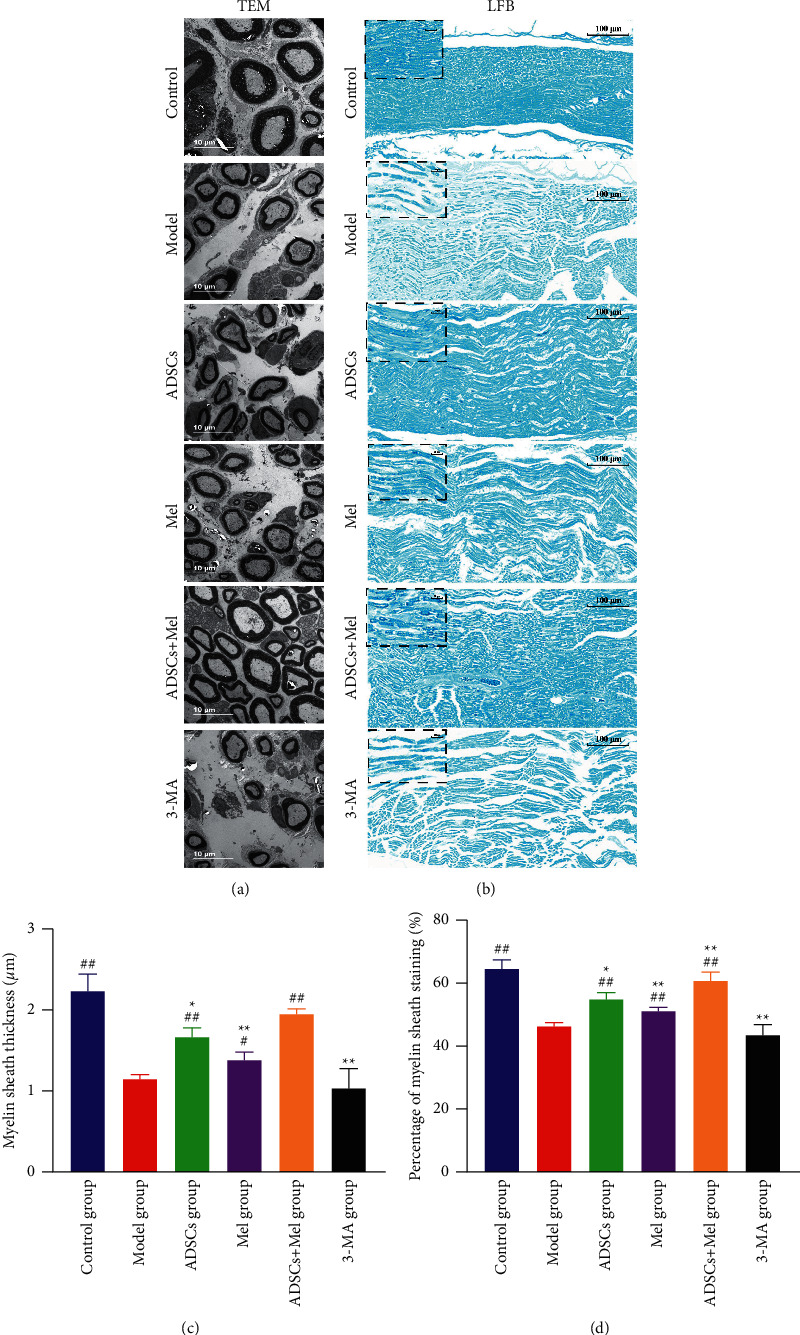
The structure of sciatic nerve in different groups. (a) TEM staining of distal sciatic nerve injury in rats. Scale bar = 10 *μ*m. (b) LFB staining of distal sciatic nerve injury in rats. Scale bar = 100 *μ*m. (c) Myelin sheath thickness in each group. (d) Percentage of myelin sheath staining. The control group, ADSCs group, Mel group, ADSCs + Mel group, and 3-MA group were compared with the model group, respectively. Each value is the mean ± SE of five independent determinations. ^##^*P* < 0.01 and ^#^*p* < 0.05, compared with the model the group;  ^*∗*^ ^*∗*^*p* < 0.05, the control group, ADSCs group, model group, Mel group, ADSCs + Mel group, and 3-MA group were compared with the ADSCs + Mel group.

To assess whether ADSCs combined with Mel can improve nerve fiber regeneration and remyelination, after 4 w of surgical operation, the histomorphology of the distal portion of the injured site was analyzed. As shown in Figure 10, the structure of the sciatic nerve of the control group stained with LFB showed uniform staining of the myelin sheaths and no evidence of demyelination. However, in the model group, the pictures showed disruption and shrinkage of myelin and swelling and atrophy of axons. In all therapy groups, this phenomenon has improved. Meanwhile, from the results of histomorphometric analysis, the percentage of stained area in the model group significantly decreased by 18.253% in comparison to the control group (*p* < 0.01). Compared with the model group, the percentage of sciatic nerve staining area in the ADSC + Mel group was significantly increased by 14.364% (*p* < 0.01), especially the percentage of stained area in the ADSC + Mel group was 5.8944% and 9.5703% higher than the ADSC group and Mel group. However, the percentage area of nerve staining dropped 17.222% extremely significantly after using 3-MA (*p* < 0.01).

We also used TEM to observe the myelin sheath thickness to evaluate the sciatic nerve regeneration. As shown in Figure 10, the TEM micrographs revealed that densely arranged myelinated fibers with thick myelin sheaths were observed in the control group. But in the model group, the regenerated myelinated nerve fibers were arranged sparsely. The myelin sheath thickness analysis showed that the average thickness of myelin in the model group (1.14 *μ*m ± 0.03) was significantly decreased by 1.06 *μ*m relative to that in the control group (2.23 *μ*m ± 0.12) (*p* < 0.01). Although some tissue fragments were still there in ADSCs, compared with the model group, the average thickness of myelin sheath in the ADSCs group, Mel group, and ADSCs + Mel group increased by 0.517 *μ*m, 0.235 *μ*m, and 0.802 *μ*m, respectively (*p* < 0.01). It is worth noting that the thicker myelin sheaths were observed in the ADSCs + Mel group, which was 0.285 *μ*m and 0.567 *μ*m thicker than that in the ADSCs and Mel groups (*p* < 0.01). However, the morphology of the myelin sheath was changed thick in 3-MA groups compared with the ADSCs + Mel group and the average thickness of the myelin sheath was markedly decreased by 0.915 *μ*m (*p* < 0.01).

### 3.6. Masson's Staining

**Figure 11:**
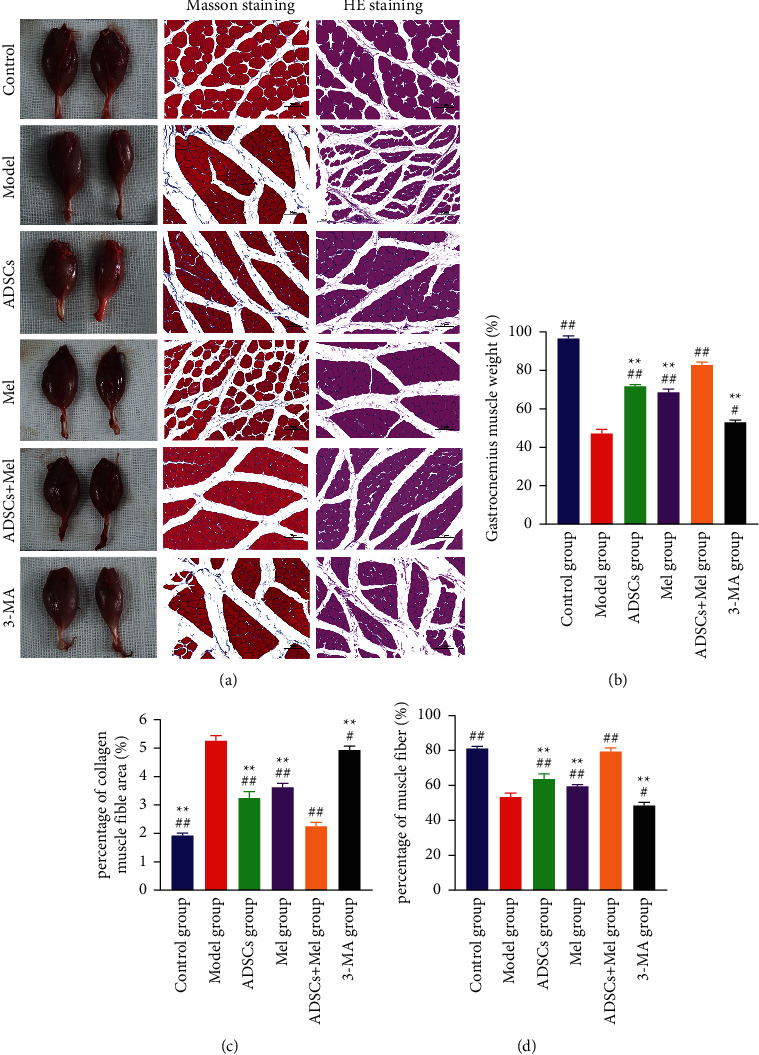
Histological assessment of the gastrocnemius muscle. (a) Image of the normal and surgical side of the gastrocnemius, Masson trichromatic staining, and H&E staining of the cross-section of the gastrocnemius. Scale bar = 50 *μ*m. (b) Gastrocnemius wet weight ratio. (c) The average percentage of collagen fiber area. (d) Muscle fiber cross-sectional area statistical results. The control group, ADSCs group, Mel group, ADSCs + Mel group, and 3-MA group were compared with the model group, respectively. Each value is the mean ± SE of five independent determinations. ^##^*P* < 0.01 and ^#^*p* < 0.05 compared with the model group;  ^*∗*^ ^*∗*^*p* < 0.05, the control group, ADSCs group, model group, Mel group, ADSCs + Mel group, and 3-MA group were compared with the ADSCs + Mel group; scale bar = 20 *μ*m.

Muscle atrophy can be eased upon reinnervation. Thus, gastrocnemius muscle samples were subjected to muscle mass measurement, H&E staining, and Masson's staining on 4 w. As shown in Figure 11, the results showed that the gastrocnemius wet weight ratio in the control group on 28 days after surgery was 96.79 ± 1.32%. Nevertheless, in the model group, the gastrocnemius wet weight ratio showed a marked decrease to 47.44 ± 1.99%. This decrease was attenuated by treatment with ADSCs, Mel, and ADSCs + Mel (68.96 ± 1.41%, 71.97 ± 0.67%, and 83.10 ± 1.23%).

Compared with control, Masson staining revealed that the atrophy of gastrocnemius muscle fibers was increased in the model group. Moreover, compared with the model group, the percentages of collagen muscle fiber area of the ADSCs group, Mel group, and ADSCs + Mel group were, respectively, reduced by 1.997%,1.618%, and 2.982%. The percentage of collagen muscle fibers area in the ADSCs + Mel group was significantly lower than that in the Mel and ADSCS + Mel groups (*p* < 0.01). The results showed a significant attenuation in the gastrocnemius muscle fibers areas in the 3-MA group compared to that in the ADSCs + Mel group.

Histologic examination of the gastrocnemius muscles showed the areas of the muscles were recorded. The results indicated that the muscle fiber area of the model group was significantly decreased by 27.462 (*p* < 0.01) compared with that of the control group. Compare with the model group, the area of muscle fibers in the ADSCs group, Mel group, and the ADSCs + Mel group increased by 10.203%, 6.017%, and 25.966%, respectively. It is noteworthy that the area of muscle fibers in the ADSCs group and Mel group was 15.763% and 19.949% more than that in the ADSCs + Mel group. Compared with the ADSCs + Mel group, the muscle fiber area of the 3-MA group was significantly decreased by 31.062%.

### 3.7. ADSCs Combined with Mel Decreases Motor Neuron Loss in Sciatic Nerve Injury

**Figure 12:**
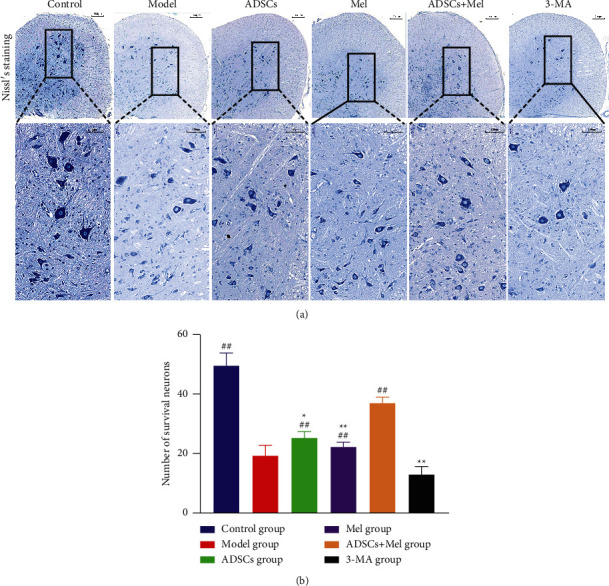
Histological assessment of the spinal cord. (a) L4 ventral horn Nissl staining. (b) The number of Nissl bodies in each group. Each value is the mean ± SE of five independent determinations. ^##^*P* < 0.01, compared with the model group;  ^*∗*^ ^*∗*^*p* < 0.05, compared with the ADSCs + Mel group.

Sciatic nerve injured may induce neuron necrosis and apoptosis in the ventral. At 4 w, after the sciatic nerve injured, Nissl staining was performed on spinal cord transverse sections to research potential neuroprotective effects of ADSCs and Mel. Nissl staining showed that (Figure 12), in a group of the model, the volume of neurocyte decreased, neurocyte had loosened cytoplasm and karyopyknosis compared with the control group. Moreover, the number of intact motor neurons was less than 30.333% within the control group (*p* < 0.01). The treatment group also increased in ventral motor neurons compared with the model group. Data display that the numbers of intact motor neurons in ADSCs and Mel groups have increased compared with the model group; meanwhile, the numbers of intact motor neurons were prominently improved in the ADSCs + Mel group compared with the model group (*p* < 0.05). Furthermore, the numbers of intact motor neurons were more in the ADSCs + Mel group than in the other two treated groups (Figure 12(b), *p* < 0.01). However, neuronal injury in the 3-MA group was dramatically more severe than that in the ADSCs + Mel group. Also consider, these data showed that ADSCs + Mel may play a critical role in neuroregeneration and autophagy has a beneficial action on PNI.

## 4. Discussion

PNI may often be damaged result of pathological or mechanical reasons. After nerve injury, the damaged axons rapidly activate the receptor tyrosine kinase erbB2 (within 10 minutes), which facilitate demyelination of SCs and dedifferentiation [30]. Then, SCs separate from axons, dedifferentiate, and secrete cytokines together with fibroblasts that prompt infiltration of immune cells [31, 32]. Subsequently, the proximal and distal of the lesion segments and the peripheral neurons and nonneural cells all react [33]. The immune cells are transformed into injured sites a few hours after being injured [34]. Then, the terminal stump of injured nerves undergoes Wallerian degeneration and leads to disruption of axonal connections. In the early stages of Wallerian degeneration (WD), damaged myelin debris or damaged organelles and malfunctioning proteins gather at the damaged site, which hinders myelin breakdown and inhibits the new myelin synthesis [31]. Therefore, rapid removal of these damaged myelin fragments or dysfunctional proteins and damaged organelles contributes to nerve regeneration.

Autophagy refers to some ingredients that are harmful to the body, such as senescent organelles and misfolded proteins, being transported for lysosomal degradation [35, 36]. The study confirmed that autophagy appeared to have two roles in peripheral nerve injury [9]. On the one hand, autophagy plays a great part in degradation of damaged organelles and digestion of misfolded proteins [37]. On the other hand, amino acids and other small molecules come into being by autophagy degradation and can be reused by the body or produce energy to protect neurons in an unfavorable nutrient environment [38]. The study found that after nerve injury, SCs will regulate their phenotype and gain the ability to clear myelin debris by enhancing autophagy [39]. In the beginning phase of myelin clearance, the SCs autophagy removes 40–50% of myelin during the first 5–7 d after injury [40]. After nerve injury, the autophagy clearance mechanism can provide basic energy and improve the injury microenvironment for SC survival [7]. Several studies have shown that autophagy has a protective effect on traumatic brain injury [41], acute spinal cord injury [42], and hypoxic-ischemic brain injury [43]. It was found that autophagy can prevent neurodegeneration of the peripheral nervous system in animal models of neuropathy [44]. Autophagy of SCs can block the occurrence and recurrence of neuropathic pain through a neural regeneration mechanism [45]. Therefore, autophagy of SCs plays a considerable role in the process of nerve repair after nerve injury. On the 7th day of nerve injury, our result showed that the expression of LC3 and Beclin-1 was increased in the model group compared with the control group (*p* < 0.01) and the number of autophagosomes in the model group also increased (*p* < 0.01).

Recovery after PNI is normally difficult and the current effective treatment is more complicated. ADSCs are easily accessible and can be induced in Schwann-like cells in vitro [46]. To sum up, compared with other stem cells, ADSCs have greater potential to promote axonal regeneration [5]. The research studies have affirmed that ADSCs can promote the regeneration of PNI and may promote nerve repair in two ways. For one thing, ADSCs secrete a wide variety of neurotrophic factors such as bFGF, TGF, *β*-1HGF, EGF, BDNF, NT-3, VEGF, and IGF-I released at different stages of regeneration, and the higher levels of neurotrophic factors may promote nerve regeneration [47, 48]. What is more, these factors are essential molecules that provide a beneficial microenvironment for neural cell survival and neurogenesis to promote the regeneration of peripheral nerves [46]. For another thing, ADSCs were able to regenerate a damaged nerve by stimulating the remaining functional cells within the neuron. ADSCs also provided a protective environment for the distal nerve end, which presented a significantly higher SC migration into the conduit [49, 50].

We injected ADSCs into the sciatic nerve injury; at the 28th postoperative day, we found that the regeneration rate of myelin in the ADSCs group was signally different from that in the model group (*p* < 0.05).

Previous studies have found that Mel, as the main hormone secreted by the pineal gland, is advantageous for the recovery of rat's sciatic nerve and crush injury. On the one hand, the study confirmed that Mel remarkably decreased sciatic nerve axonal injury and oxidative stress after injuries [51]. After body injury, free oxygen, free radicals, and many toxic substances accumulate around the injury site. This interferes with cell membrane permeability and stimulates intracellular calcium influx. Intracellular calcium influx in turn activates the proteolytic pathway, leading to cellular destruction, including destruction of nerve filaments and microtubules [52]. It has been found that Mel can restore cellular metabolic function and improve Ca^2+^ signal transduction [53]. On the other hand, Mel can shorten harmful results of free radicals by motivating antioxidant enzymes and damping posttraumatic polymorphonuclear infiltration [54]. Our results also revealed the good impact of Mel on sciatic nerve recovery. Rats treated with Mel gave evidence of better structural regeneration of the myelin sheaths compared to the model group (*p* < 0.05). In addition to this, Mel has been displaying to play a part in regulating the migration, proliferation, apoptosis, and differentiation of stem cells [55]. Studies have found that Mel's immediate detoxification of reactive oxygen species or incentive antioxidant enzymes can protect stem cells resistant to oxidative stress injury [19, 56]. Additionally, Mel promotes human adipose-derived mesenchymal stem cell proliferation by preventing cell replication senescence [57]. While, Mel also promotes secretion of proangiogenic/mitogenic factors, providing a good microenvironment to influence stem cells' survival [58]. The results of these studies have confirmed that Mel pretreatment may enhance the efficacy of stem cells in treating diseases by enhancing the self-renewal potential of stem cells, promoting stem cell proliferation, and reducing the expression of proinflammatory factors, providing a good microenvironment for stem cells.

Considering that Mel does not directly enter the damaged area after incubation of stem cells with Mel [19], Mel may not put a long-lasting protective influence on stem cells to cope rugged microenvironment due to the inflammatory microenvironment and oxidative stress in nerve tissue [59]. Thence, finding a long-lasting and convenient way to improve stem cells combined with Mel to promote nerve axonal regeneration is essential. In addition to this, several studies confirmed that only the use of Mel has a beneficial effect on the regeneration of myelin sheaths in PNI [60, 61]. Therefore, we use ADSCs combined with Mel to inject the lesion to treat the peripheral nerve injury. After 28th, our study shows that the use of Mel combined with ADSCs can effectively improve the effect of nerve repair. In the early stage of nerve injury, damaged tissue is quickly cleared by autophagy; therefore, we used autophagy inhibitor 3-MA to evaluate the autophagy activity in the early stage after transplantation of ADSCs combined with Mel. On the 7th day after surgery, we found that the ADSCs combined with the Mel group had increased protein expression levels of LC3 and Beclin-1 compared to the use of only adipose stem cells or Mel (*p* < 0.05). The number of autophagosomes in the adipose stem cells combined with the Mel group also increased significantly, which is consistent with the WB results. Conversely, inhibiting autophagy using 3-MA adenine strongly inhibited the formation of autophagosomes. On the 28th day after surgery, we found that the ADSCs combined with Mel group recovery were better than those using 3-MA.

Consequently, we have not only observed the effect of the combination on nerve regeneration but also selected autophagy inhibitors 3-MA under the lesion model of sciatic nerve injury. According to the data, study the impact of role of autophagy in peripheral nerve regeneration after sciatic nerve injury in rats. In summary, after treatment with the autophagy inhibitor 3-MA, various indexes of nerve regeneration were significantly reduced, confirming that ADSCs combined with Mel can promote sciatic nerve regeneration in rats by changing the autophagy activity in the early stage of sciatic nerve injury.

## Data Availability

The data used to support this study are available from the corresponding author upon request.

## Abbreviations

ADSCs: Adipose-derived stem cells
PNS: Peripheral nervous system
SCs: Schwann cells
MSCs: Mesenchymal stem cells
S-100: Soluble protein-100
MBP: Myelin basic protein
NF-200: Neurofilament
3-MA: 3-Methyladenine.
